# Growth factors-based therapeutic strategies and their underlying signaling mechanisms for peripheral nerve regeneration

**DOI:** 10.1038/s41401-019-0338-1

**Published:** 2020-03-02

**Authors:** Rui Li, Duo-hui Li, Hong-yu Zhang, Jian Wang, Xiao-kun Li, Jian Xiao

**Affiliations:** 1 Molecular Pharmacology Research Center, School of Pharmaceutical Sciences, Wenzhou Medical University, Wenzhou, 325035 China; 2 School of Chemistry, Sun Yat-sen University, Guangzhou, 510275 China; 3 Department of Peripheral Neurosurgery, The First Affiliated Hospital, Wenzhou, Medical University, Wenzhou, 325000 China

**Keywords:** growth factors, peripheral nerve injury, nerve conduits, signaling cascade, axonal regeneration, nerve growth factor, basic fibroblast growth factor

## Abstract

Peripheral nerve injury (PNI), one of the most common concerns following trauma, can result in a significant loss of sensory or motor function. Restoration of the injured nerves requires a complex cellular and molecular response to rebuild the functional axons so that they can accurately connect with their original targets. However, there is no optimized therapy for complete recovery after PNI. Supplementation with exogenous growth factors (GFs) is an emerging and versatile therapeutic strategy for promoting nerve regeneration and functional recovery. GFs activate the downstream targets of various signaling cascades through binding with their corresponding receptors to exert their multiple effects on neurorestoration and tissue regeneration. However, the simple administration of GFs is insufficient for reconstructing PNI due to their short half‑life and rapid deactivation in body fluids. To overcome these shortcomings, several nerve conduits derived from biological tissue or synthetic materials have been developed. Their good biocompatibility and biofunctionality made them a suitable vehicle for the delivery of multiple GFs to support peripheral nerve regeneration. After repairing nerve defects, the controlled release of GFs from the conduit structures is able to continuously improve axonal regeneration and functional outcome. Thus, therapies with growth factor (GF) delivery systems have received increasing attention in recent years. Here, we mainly review the therapeutic capacity of GFs and their incorporation into nerve guides for repairing PNI. In addition, the possible receptors and signaling mechanisms of the GF family exerting their biological effects are also emphasized.

## Introduction

Peripheral nerve injury (PNI) is a very common chronic trauma seen in clinics and is characterized by permanent sensory impairment and severe motor dysfunction [1]. A long period of denervation may worsen muscle atrophy and lead to a decline in quality of life. Statistically, the number of PNI diagnoses caused by accidents or trauma each year is ~200,000 in the United States and 300,000 in Europe [2]. Although peripheral nerve fibers show considerable potential for self-regeneration, the outcome is generally unsatisfactory, resulting in weak motor recovery and irreversible sensory dysfunction.

According to the extent of the severity after injury, peripheral nerve damage is classified into five categories, as described by Sunderland [3]. Grade I is defined by neurapraxia and focal demyelination; grade II displays axonotmesis but an intact neuronal stroma; grade III involves loss of the funiculus and its contents; grade IV entails disruption of all portions of the nerve; and grade V involves the entire nerve trunk. For nonsevere injuries (grades I–III), the adult nerve itself has a certain intrinsic regenerating capability. Patients only need exercise training and physical therapy as treatment. For grade IV and V injuries, the nerve gaps require suturing via surgical techniques, including manipulative nerve operations and bridge operations [4]. However, the connection-site morbidities, such as peripheral neurofibroma formation and fiber shape mismatch, of those operative interventions often lead to unsatisfying results [5]. With the development of tissue engineering technology, several nerve scaffolds/conduits that support neural regeneration over large nerve gaps potentially serve as an alternative method for reinnervating severe peripheral nerve defects [6]. Many of these nerve scaffolds/conduits possess proper biocompatibility and mechanical strength that protect injured neurons and Schwann cells (SCs) from apoptosis and prevent the formation of scars [7]. However, some unknowns exist, such as the suboptimal effectiveness, that limit the availability of these scaffolds/conduits for clinical use [8]. To optimize their properties, increasing research efforts have focused on biomimetic neural scaffold/conduit fusing growth factors (GFs) to repair PNI and achieve superior therapy for promoting nerve regeneration and functional reinnervation [9–11].

GFs are polypeptides that support cell survival, proliferation, differentiation, and morphogenesis in the mammalian nervous system [12]. These beneficial effects mainly occur through strengthening the intrinsic transduction potentiality, regulating interaction with molecular and mechanical signaling cascades after receptor-mediated retrograde uptake [13, 14]. The GF family members, including nerve growth factor (NGF) and fibroblast growth factors (FGFs, containing FGF_1~23_), are secreted prominently by SCs or neurons under physiological conditions. In the peripheral nervous system (PNS), GFs maintain the regenerating microenvironment for axonal elongation and sprouting after traumatic nerve injury. In the peripheral nervous system, the role of GFs in neuronal survival and the capacity for neurite outgrowth has been extensively researched [15]. Further studies revealed that GF expression was upregulated in injured distal nerve stumps. Unfortunately, endogenous GFs did not satisfy the demand for axonal myelination and nerve outgrowth [16]. In these studies, exogenous GFs were required to be administered continuously over a long period of time to provide trophic support for axon regeneration.

To achieve the goal of axonal regeneration, numerous studies have employed GF loading into neural scaffolds/conduits to enhance the speed and accuracy of injured nerve restoration [17]. For instance, glial-derived neurotrophic factor (GDNF) supplementation of multiluminal conduits had a beneficial effect on inducing axon outgrowth and target reinnervation compared to GDNF-free conduits in a 4 cm nerve gap injury [18]. NGF, brain-derived neurotrophic factor (BDNF) and insulin-like growth factor (IGF) encapsulation into gelatin-based nerve guidance conduits (NGCs) showed promising potential for neurite guidance and extension, as well as functional recovery [19]. Compared with GFs or the application of grafted biomaterial alone in repairing PNI, combining them together has several advantages. These advantages include (1) the protection of GFs from enzyme digestion and the prolongation of their bioactivity; (2) the localization of GFs at the target site and the control of their release for a sufficient period of time; (3) the interaction between multiple GFs and GF delivery system, which could provide a synergistic effect on accelerating neurite outgrowth and elongation; and (4) the creation of a better regenerative microenvironment and linear order guidance for achieving appropriate target reinnervation and functional connection. Currently, scientific researchers prefer to deliver multiple GFs rather than only one kind of GF to treat PNI due to their coordinated interactions with each other [20].

A crucial tenet of acquiring beneficial therapies for peripheral nerve regeneration is the administration of a high dose of GFs (ranging from several micrograms to dozens of milligrams) [21]. Nevertheless, this approach raises concerns regarding the GF duration, dosage or immune response when they enter into the mammalian body. To solve this issue, current knowledge combines various controlled delivery systems with GFs, such as direct incorporation, layer-by-layer technology or multiphase loading methods, which allow GF release in a sequential and spatiotemporal fashion, leading to GFs being retained by the region of interest with a desirable concentration [17, 22]. In addition, the biocompatibility of GF-based delivery systems also needs to be considered because this determines whether they can be applied in clinical practice. In the last few decades, the materials utilized in the preparation of different types of GF-loaded nerve scaffolds/conduits have been roughly classified into two types: nondegradable materials and biodegradable materials. Nondegradable materials include silicone, poly-lactide (PLA), poly-*l*-lactide (PLLA), and poly (*D*, *L*-lactide-co-glycolide) (PLGA). They are bioinert materials and seldom elicit any undesirable responses in the eventual host. However, mostly biodegradable materials are derived from living donors, fresh corpses or animals. Undesired residual components, such as glycosaminoglycan, endotoxins, and host rejection proteins (α-gal epitope and MHC-1), inevitably invoke implant rejection and a host response when they are implanted in vivo [23]. The utilization of acellular technology, including mechanical, enzymatic, and chemical means, can remove many immunogenic components, but large amounts of the extracellular matrix (ECM) components are inevitably dislodged as well [24]. Thus, the preparation of natural nerve scaffolds/conduits is required for maximum retention of ECM composition and for the greatest possible removal of undesired immunogenic components.

In this review, we first provide a discussion of the complex process of the intrinsic pathological changes after PNI. Then, we discuss the potential effects of different GFs and GF-loaded delivery systems on peripheral nerve reconnection/reconstruction and reveal the related signaling mechanisms. Ultimately, we selected NGF and FGF-2 as typical examples for the GF family to systematically describe their effects on the development and function of injured nerves.

## Intrinsic regenerative and degenerative responses to PNI

**Fig. 1:**
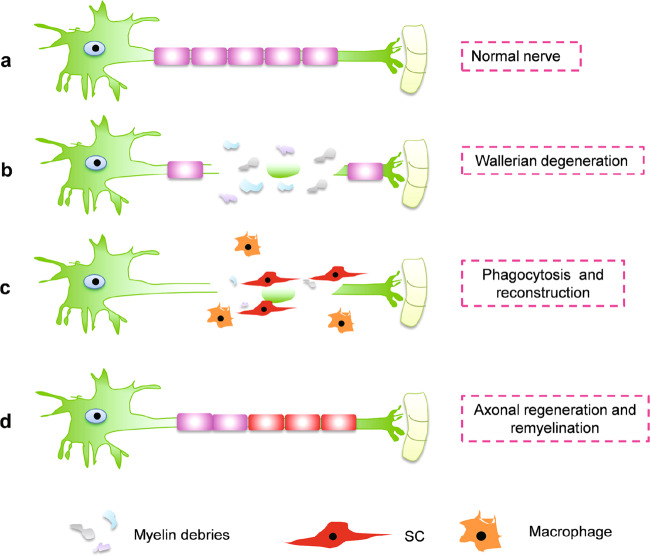
Schematic diagram illustrating the process of degeneration and regeneration after PNI. When normal nerves (**a**) suffer from physical injury, the portion of the lesion site and its distal stump undergo destruction and breakdown and produce myelin debris. This degenerative process is called WD (**b**). Then, SCs recruit macrophages to scavenge degenerated myelin fragments (**c**). Meanwhile, SCs proliferate and migrate alone the basal lamina to form bands of Büngner, which guides axon to reinnervate towards the corresponding target (**d**).

Traumatic PNI is generally characterized by neurapraxia, axonotmesis and neurotmesis [25]. Regardless of the kind of injury, the distal stump of the damaged peripheral nerve initiates a complex series of several cellular and molecular changes, known as Wallerian degeneration (WD) [26]. WD can be generally divided into an early stage (up to 5 days) and a later stage (5–14 days) [27]. During the early stage, the damaged region and its distal end are separated from the corresponding proximal nerve trunk and undergo large morphological changes, including granular disintegration of the cytoskeleton, the formation of ovoid-like myelin structure and fragmentation indicative of degeneration [28]. Cleaning up the axonal fragmentation and myelin debris may contribute to a favorable regenerative microenvironment for nerve regeneration [29]. Hence, at the later stage, surviving SCs undergo dedifferentiation and recruit the surrounding macrophages to engulf and digest myelin lipids and other necrotic tissues in the distal nerve area. At the same time, dedifferentiated SCs begin to proliferate and migrate along the basal lamina tube to form the band of Büngner (Fig. 1). During this process, SCs also produce and release different GFs to create a permissive growth environment for guiding the growth cone towards the denervated target organs. Ultimately, proliferative SCs undergo dedifferentiation and transform their phenotype to ensheath the large diameter of immature axons by forming consecutive layers of plasma membrane [30, 31]. The regenerated myelin sheaths regain rapid signal transduction along the dominated target organization by modulating their inner ion channels and neurotransmitter transport [32]. Thus, plastic SCs play a vital role in structural and functional recovery after PNI.

Peripheral nerves have a certain growth capacity for self-healing. The present research indicates that this self­repair and intrinsic growth capacity largely depend on SC-axon interactions, myelin clearance, blood supply and GF supplementation [33, 34]. Interestingly, direct interaction between SCs and axons seems to be of pivotal importance for axonal elongation and remyelination [35]. Their interaction occurs mainly through the secretion of some cytokines. On the one hand, once growing axons contact the SC surface, they secrete neuregulins from their growth cones to enhance SC proliferation, migration and myelination. On the other hand, proliferating SCs provided trophic factors (including GFs) and surface proteins to guide regenerative axon growth towards the distal stump [36]. This intrinsic cooperative SC-axon interactive communication provides a new point of view for understanding the mechanisms underlying axonal regrowth and target reinnervation during the whole-nerve regeneration process.

Macrophages are also known to play multiple roles in the process of peripheral nerve regeneration. When macrophages infiltrate injured nerves, they positively affect myelin destruction and removal through opsonin-dependent and opsonin-independent mechanisms [37]. Moreover, this time course of myelin debris clearance is overtly seen starting from 1 to 4 days, peaking at 7 days and lasting for 14 days after the injury [38]. Mechanistic studies have demonstrated that the degree and speed of myelin removal are determined by the kinetics of macrophage recruitment and the cooperation of the macrophage-SC in interacting to scavenge degenerated myelin [39]. In addition to myelin phagocytosis, macrophages also secrete multiple cytokines and chemokines [40]. Some of them, such as GFs, interleukin-1, and apolipoprotein E, facilitate SC and neuronal survival, axonal elongation and peripheral nerve reconstruction as well as an anti-inflammatory response. Other cytokines, including tumor necrosis factor α (TNFα), tumor growth factor β (TGFβ) and leukemia inhibitory factor (LIF), cause a pro-inflammatory response. If this proinflammatory reaction is excessive or prolonged, there is an unreasonable accumulation of scar-associated molecules, such as chondroitin sulfate proteoglycans (CSPGs), which impede axonal elongation and neurite outgrowth [27]. Thus, the regulation of inflammation is crucial for axonal regeneration and remyelination following nerve trauma.

Neuronal survival, axonal outgrowth, and synapse formation during nerve regeneration require the combined action of multiple intrinsic and extrinsic factors. The functions of these factors can be summarized as follows: (a) the modification of the regenerative microenvironment, which can occur through inhibiting the inflammatory factor expression, reducing oxidative stress levels and accelerating myelin fragment clearance; (b) the supplementation of GFs at the cell soma and axon tips; (c) the activation of intrinsic growth programs of injured nerves via increasing intracellular transcriptional factors; (d) the prevention of neurofibroma formation at the lesion site to guide regenerated axons to connect with their original targets; and (e) the balance between growth-promoting and growth-limiting molecules, such as trophic factors, neuregulin 1, CSPGs, and myelin-associated glycoprotein. Thus, focusing on the correlative factors involved in nerve regeneration can guide us in seeking a promising therapeutic strategy for the treatment of PNI.

## The multiple roles of GFs in peripheral nerve reconstruction

GFs belong to the neurotrophic factor family of peptides that play critical roles in controlling survival, proliferation, migration and differentiation in developing and regenerating nerve fibers. Members of the GF family, including NGF, the FGF family, neurotrophin-3 (NT-3), BDNF and platelet-derived growth factor (PDGF), are mainly secreted from the nerve system and other organic tissue throughout the whole developmental progress [41]. The preclinical or clinical applications of these factors in treating different kinds of diseases are shown in Tables 1 and 2.

**Table 1:** GFs employed in pre-clinical trials.

Name	Advantage	Treating diseases
NGF	• Essential for neural regeneration and neurotrophic function	Diabetic peripheral neuropathy [139] Peripheral nerve injury [124]
FGF1	• Classic mitogenic and neuroprotective activities	Diabetes mellitus [140] Diabetic nephropathy [141]
FGF2	• Angiogenesis • Stimulating cell proliferation, migration, and differentiation	Wound healing [142] Diabetic foot [143] Spinal cord injury [144]
FGF21	• Facilitating glucose and lipid intake • Improving insulin sensitivity • Metabolic regulator	Obesity [145] Hyperlipidemia [146] Alcohol-induced hepatic steatosis [147]
NT-3	• Regulation of axonal and dendritic outgrowth, synapse formation and function • Promoting neuronal survival Enhancing neurite growth and axonal regeneration	Traumatic brain inury [148] Spinal cord injury [149]
BDNF	• Synapsis plasticity • Neuronal survival • Formation of new synapses	Depression [150] Metabolic disorders [151]
PDGF	• Modulation of neurogenesis, cell survival and synaptogenesis	Tumorigenesis [152] Alzheimer disease [153]

**Table 2:** Part of GFs employed in clinical trial or application.

Name	Type of diseases	Dosage	Outcomes	Ref.
NGF	Chronic ulcerations	50 μL/day (200 μg/mL) for 4–6 weeks	• Accelerating wound healing of the chronic cutaneous ulcer	[154]
			• Inducing angiogenesis	
	Diabetic neuropathy	0.1 μg/kg rhNGF subcutaneously three times a week for 6 months	• protecting peripheral nervous system neurons and normalizing their activity • Causing acute pain reaction	[155]
FGF1	Spinal cord injury	combination FGF1 and fibrin glue (2 mL) at 3 and 6 months postsurgery via lumbar puncture.	• Improving ASIA motor and sensory scale scores • Increasing the intrinsic activity of neurons	[156]
FGF2	Type 2 diabetes mellitus	0.5 μg/kg per day FGF1 for 16 weeks	• Improving glucose sensing and uptake • Enhancing insulin sensitivity	[157]
	Acute stroke	Patients were intravenously administered FGF2 ranging from 3 to 150 μg/kg for 24 h	• Significant reducing intracerebral hemorrhage • A mild advantage on decreasing mortality rates	[158]

Following peripheral nerve damage in normal adult rodents, SCs in the injured area switch from a transmitter state to a regenerative state and initiate the transcription of GF mRNA, subsequently inducing the upregulation of GF protein expression through an autocrine or a paracrine manner [42]. Moreover, the GFs expressed basically present a bell-shaped trend; namely, there is elevated expression in the early phases of injury that changes to lower levels of expression within a month of repair [43]. The extent of recovery of motor and sensory function is closely related to this complicated trend of different GF expression [44]. When the expression of endogenous GFs declines, cellular metabolic balance and energy supply will not be maintained in SCs and neurons, which seriously affects the regenerative ability of the impaired nerve after long-term denervation [45]. Thus, the addition of exogenous GFs has become a promising strategy for repairing PNI.

GFs exert their biological effects and signal transduction activities through interaction with their specific receptors. Currently, the mammalian GF family can be subdivided into three subfamilies, namely, canonical growth factors (cGFs), intracellular growth factors (iGFs), and hormone-like growth factors (hGFs). All GFs present a heparan sulfate proteoglycan (HSPG) binding domain, which binds to a set of membrane-bound growth factor receptors and activates their signal transduction via affinity for β-Klotho [46]. The cGF subfamily contains the most GFs, including NGF, FGF1-10, NT-3, and BDNF. These GFs contain an N-terminal signal peptide (SP), which is correlated with the interaction of the tyrosine kinase receptors (TrkA, TrkB, and TrkC) with extracellular molecules in an autocrine or paracrine manner. TrkA is a single-pass transmembrane protein that is regarded as a high-affinity receptor for NGF and is primarily expressed in primary sensory neurons. TrkB and TrkC, which are high-affinity receptors for BDNF and NT-3, are normally found in spinal motoneurons and in large-diameter primary sensory neurons [47]. It is important to emphasize that FGF1-10 can interact with specific FGF receptors (FGFR1-4) that belong to the protein tyrosine kinase (PTK) family. iGFs (including FGF11 to 14) are also called FGF homologous factors (FHFs). They are expressed predominantly in intracellular membranes, and they interact with the intracellular domains of voltage-gated ion channels rather than activating FGF receptors. The third subset of FGFs is called hGFs (consisting of FGF15/19, FGF21, and FGF23). hGFs are produced from the liver and adipose tissue and serve as endocrine factors to regulate metabolism at a long distance. Furthermore, it is important to consider that the p75-neurotrophin receptor (p75^NTR^, 75 kDa), which is a low-affinity receptor for NGF, is widely expressed in SCs and is markedly upregulated during the axon elongation phase [48]. The role of p75^NTR^ in eliciting either anti- or proapoptotic signaling relies on the cellular environment and tissue state [49, 50]. Knowing which GFs operate and interact with their specific receptors is very important for understanding the downstream signaling cascades that mediate cytoskeletal rearrangements and axonal outgrowth in the adult mammalian PNS.

GFs are polypeptide regulatory molecules that consist of two pairs of antiparallel β-chains that interact through noncovalent binding [51]. They have approximately 50% sequence homology due to their highly conserved structural features [52]. Several studies have confirmed that GFs persistently facilitate isolated neuronal survival and outgrowth in vitro. The multiple roles of GFs in vivo have been adequately demonstrated and include promoting SC migration, axonal regrowth, remyelination and peripheral target reconnection during nerve rehabilitation (therapeutic GFs with peripheral nerve regeneration capabilities in rodent models are shown in Table 3) [53–55]. However, during the postnatal development and early regeneration periods, the damaged neurons and their distal nerve stump have insufficient GFs to support their growth and regeneration [43]. Long periods of inadequate GF supply will increase neuronal apoptosis and death. Therefore, the administration of exogenous GFs to support axonal and myelin regeneration will become an important therapy for treating acute PNI. A large amount of research has demonstrated that NGF noticeably promotes axonal regeneration and increases electrophysiological parameters and behavioral recovery following PNI [56–58]. Another kind of GF, FGF-2 (also called bFGF), is strongly expressed in the dedifferentiated SCs of intact damaged nerves, which exhibit similar biological activities for the regeneration of peripheral nerve defects as what is seen in functional, morphological, and histological evaluations [59, 60]. Local infusion of BDNF increases the size and myelin thickness of regenerating axons in a dose-dependent manner when applied in an adult rat sciatic nerve transected model [61, 62]. Previous studies showed that elevated NT-3 had a trophic role in enhancing the regenerative potential of lesioned nerves and the reinnervation of denervated target organs [63, 64]. Accordingly, the inhibition or blockade of these GF supplements may reduce axonal sprouting and affect the reinnervation ability after nerve lesions.

**Table 3:** Therapeutic potential of GFs for peripheral nerve regeneration.

Name	Drug administration and dosage	Animal models	Outcomes	Ref.
NGF	Administration of 80 ng NGF/day for 3 weeks via via T-tube nerve chambers	Crush injury approximately 4–5 mm in rats	• Superior axonal regeneration and robust behavioral locomotor recovery	[124]
FGF1	aFGF mixture (2.1 mg/mL) was placed in the gap	5-mm sciatic nerve gap in rats	• Creating a favorable environment for axonal regeneration and locomotor function	[159]
FGF2	Scafford incorporated with bFGF (400 μg) via bridging the proximal and distal nerve stumps	35-mm facial nerve gap in minipig	• Elevating electrophysiology and histomorphological parameters • Improving motor and sensory impairments • Suitable for long nerve gap repairing	[138]
FGF21	Intramuscular injection of FGF21 (50 μg for each rat) at 24-h intervals for 7 days	Crush injury with two vascular clips at 2 mm intervals	• Ameliorating motor and sensory function • Enhanced axonal remyelination and regrowth • Accelerating Schwann cells (SCs) proliferation	[86]
NT-3	The nerve ends were grafted with fibronectin mats impregnated with NT-3 (500 ng/mL)	1-cm sciatic nerve defects in adult Lewis rats	• Enhancing nerve regeneration • Sustainable increasing myelinated fibers	[160]
BDNF	The nerve gap was bridged by the fascial tube filling with BDNF (600 μg)	2-cm sciatic nerve defect in SD female rats	• Increasing nerve fiber growth • Inducing faster nerve regeneration • Relieving neuropathic pain	[161]
PDGF	20 μL PDGF solution (0.5 ng/mL) was filled in the silicone conduit	10-mm sciatic nerve gap in rats	• Improving locomotion recovery • Accelerating nerve regeneration •Facilitating Schwann cells migration	[162]

Because GFs are protein drugs, they easily undergo proteolytic degradation and are quickly inactivated in a normal-temperature environment. Moreover, because GFs rapidly diffuse in the body fluids, there is an eventual inadequate GF concentration in lesion sites [65]. Additionally, the short half-life of GFs requires their exogenous application at continuous and high doses to achieve an optimal therapeutic concentration. Thus, selecting or designing an adaptable delivery vehicle that persistently maintains GF bioactivity and controlled release in a steady fashion will be the optimal choice for nerve regeneration. NGCs as potential alternatives to autologous nerve grafts have been approved by the U.S. Food and Drug Administration (FDA) for repairing peripheral nerve gaps of less than 20–25 mm. These conduits are composed of biodegradable and nonbiodegradable materials [66], such as NeuraGen® (Integra), NeuroMatrix®/Neuroflex® (Stryker), Neurotube® (Synovis), SaluBridge® and SaluTunnel® (SaluMedica), and Neurolac® (Ascension). They all possess good biocompatibility and the appropriate mechanical strength to support SC growth, proliferation and migration. Thus, NGCs loaded with specific GFs may represent an effective therapeutic tool for peripheral nerve defects. A promising biodegradable material, poly(ethylene arginyl aspartate diglyceride) (PEAD), conjugated with NGF or FGF-2, promoted structural and functional recovery as well as enhanced connections between immature SCs and axons [67, 68]. The addition of immobilizing NGF onto chitosan or polycaprolactone (PCL), producing nerve conduits, was confirmed to successfully connect the proximal and distal nerve ends in a rat sciatic nerve defect model [69, 70]. Incorporating bFGF into collagen scaffolds was suitable for guiding the direction of nerve reconstruction and appropriately increased the weight of the gastrocnemius muscle for a peripheral nerve defect injury [71]. The local delivery of BDNF through collagen-based conduits has been shown to reduce the initial burst of GF release and to provide a regenerative microenvironment for impaired peripheral nerve reconstruction [72]. All of those studies indicate that the combination of GFs and NGCs may hold promise for enhancing therapeutic efficacy post injury.

However, peripheral nerve regeneration is a dynamic process that is regulated by numerous GFs that coordinate with each other to achieve the ideal functional restoration of damaged or defective nerves. Single-GF supplementation may not be sufficient for promoting different stages of nerve regeneration and supporting different neuronal subpopulations that rely on different GFs [20, 73]. The delivery of multiple GFs via carrier biomaterials in a specific spatial and temporal manner to imitate the internal environment of the body is regarded as a better promising strategy for GF-based therapeutics than many previous approaches. This technological strategy for boosting neurite outgrowth seems to be useful for the repair of peripheral nerves [20]. It has been demonstrated that collagen nerve conduits loaded with GDNF and NGF synergistically accelerated the rate and quality of early nerve structural regeneration in terms of newborn axonal numbers and proliferative SC states in a severed rat sciatic nerve gap model [73]. Silk nanofiber codelivery of NGF and ciliary neurotrophic factor (CTNF) may be suitable for simulating neurite outgrowth and guiding glial cell migration in terms of neural responses [74]. Our team incorporated FGF-2 and NGF into a novel thermosensitive heparin-poloxamer (HP) hydrogel to form a GF-HP hydrogel. The results showed that this GF-HP could facilitate SC proliferation, enhance axonal regeneration and remyelination, and improve the recovery of motor function in the PNI of diabetic rats [75]. Regardless of which GFs are used for PNI repair and whether or not they are associated with biomaterials, the regenerative efficiency mainly depends on GF action [72]. Thus, we selected GFs as examples to clarify their molecular mechanisms in regulating axonal regeneration and target reinnervation.

## GF signaling mechanisms that regulate nerve regeneration

**Fig. 2:**
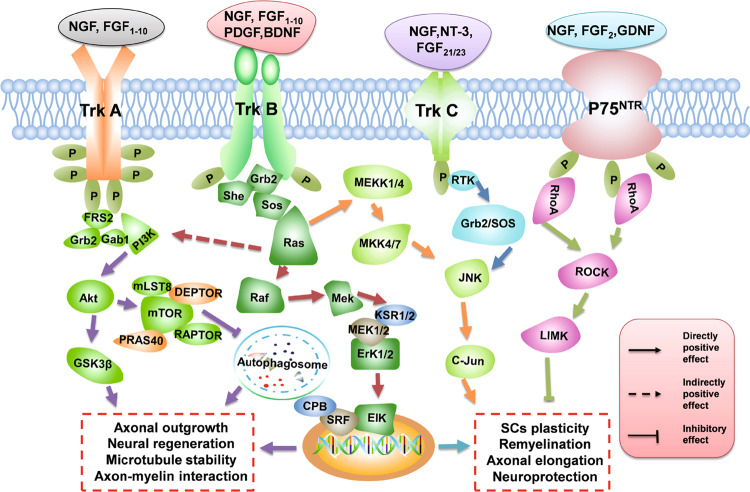
Schematic representation of the typical GF signaling cascade and its main downstream effectors. When GF ligands are bound to their corresponding membrane spanning receptors (TrkA, TrkB, TrkC, P75^NTR^), they become phosphorylated and interact with other adaptor proteins, such as FRS2, Grb2, Gab1, She, Grb2, or Sos, to form one of various arrangements of multiprotein complexes that localize to the cell membrane and activate downstream signaling cascades, including PI3K/Akt, MAPK/ERK, JNK/c-Jun, and Rho A/ROCK. These cascade activation events are implicated in axonal outgrowth or regeneration, SC plasticity, remyelination, microtubule stability and neuronal survival following nerve injury.

The enhancement of axon growth and neurite outgrowth by GFs is regulated by diverse signaling mechanisms, including phosphatidylinositol-3 kinase/protein kinase B (PI3K/Akt), mitogen-activated protein kinase/extracellular signal-related kinase (MAPK/ERK), c-Jun N-terminal kinase (JNK)/c-Jun, RhoA/ROCK and other types of noncanonical signal transduction pathways (classic and nonclassic signaling cascades of GFs exerting their biological actions are presented in Figs. 1 and 2, respectively) [76, 77]. These signaling transduction pathways are activated at different stages of nerve regeneration through retrograde axonal transport. This retrograde transport occurs mainly through directly modulating the activities of dynein-dynactin in the neuronal cytoskeleton, which transport organelles, proteins, and RNA from the distal axon to the soma [78]. After the administration of exogenous GFs to the target site, biological GFs begin to bind their corresponding receptors on the cell membrane to initialize the local translation of gene expression, resulting in the activation of intrinsic downstream signaling pathways. This series of signaling cascades finally lead to microtubule reorganization, growth cone remodeling, local protein synthesis and specific membrane restructuring. Their specific regulatory processes are elaborated below.

### PI3K/Akt signaling

**Fig. 3:**
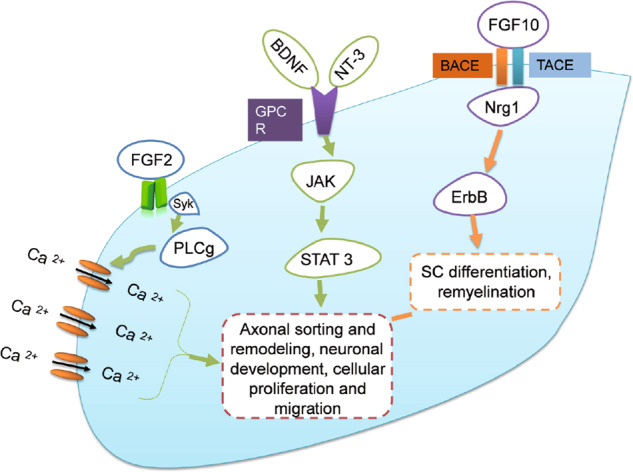
Other signaling cascades that involve GFs controlling PNI repair. Some GFs, such as FGF10, BDNF, NT-3 and FGF-2, coordinately activate the Nrg1/ErbB, JAK/ STAT 3, or PLCg/Ca^2+^Oct6 pathways to initiate neuronal development, axonal remodeling, SC differentiation and remyelination.

The PI3K/Akt pathway commonly contributes to many areas of physiology and pathology, including cellular growth, survival, and metabolism Fig. 3. In mammalian PNS, GFs perform their biological actions of promoting intrinsic neurite outgrowth and synaptic plasticity by working together with PI3K/Akt signaling [79]. Furthermore, previous research has demonstrated that NGF-induced PI3K/Akt activation occurs through binding the TrkA receptor rather than p75^NTR^ to promote neurite outgrowth and neuronal survival in cultured primary neurons [80]. Suppressing this pathway with LY294002 (a special PI3K inhibitor) can significantly influence the effects of NGF on the regulation of neurite survival and axon outgrowth [81, 82]. It should be noted that the activation of PI3K/Akt signaling also triggers downstream components, such as mammalian target of rapamycin (mTOR) and glycogen synthase kinase 3β (GSK3β), which enhance cell proliferation, migration and differentiation. Increasing evidence has confirmed that the PI3K/Akt/mTOR and PI3K/Akt/GSK3β signaling pathways are closely associated with growth cone collapse, neuroplasticity and axonal extension in the developing and regenerating nervous system [83]. Moreover, our team and other researchers have found that some GFs, including NGF, FGF-2 and FGF21, could activate either or both of these pathways to suppress the effects of different extracellular stimuli, such as oxidative stress, endoplasmic reticulum (ER) stress or inflammation, creating a favorable microenvironment for nerve regeneration [84–86]. Recently, Geary and Ness et al. have revealed that IGF-1 and NT-3 can strongly induce axonal branching and turning through enhancing the phosphorylation of PI3K and Akt at the leading edge of the growth cone [83, 87]. Thus, GF-mediated PI3K/Akt activation is associated with substantial benefits following PNI.

### MAPK/ERK signaling

MAPK/ERK also acts as an important regulator for neuronal protection and commitment. Following axotomy, the MAPK molecule binds to threonine 202 and tyrosine 204 of ERK and phosphorylated ERK, which leads to activated ERK translocation into the nucleus to trigger a regenerative response in the PNS [88]. By utilizing NGF or NT-3 to strengthen MAPK/ERK signaling, SCs or normally oligodendrocyte-ensheathing axons may be induced to form compact myelin wraps. Moreover, the activation of the MAPK/ERK pathway has also been shown to contribute to spontaneous neurite outgrowth and axon elongation in dissociated DRG cultures [89]. In contrast, the inhibition of ERK phosphorylation significantly delays axotomy-induced growth cone formation and axon regrowth in denervated muscle fibers [90]. Similarly, knockdown of the MAPK gene significantly weakens the potential regenerative effects of FGF21 on axonal regeneration, remyelination, and functional recovery following facial nerve injury [91, 92]. Therefore, clarifying the roles and actions of GFs interacting with the MAPK/ERK pathway may provide a better understanding of the molecular mechanism underlying the action of GFs in PNI repair.

### JNK/c-Jun signaling

JNK/c-Jun is another important signaling pathway that controls neuronal survival, axon plasticity and remyelination [93]. Parkinson et al. showed that JNK-mediated axon regeneration and remyelination were driven by MAP kinase kinase 7 (MKK 7), suggesting that MKK 7 is upstream of the JNK/c-Jun pathway in regulating neuronal survival and axonal regrowth. The JNK/c-Jun pathway also triggered the activation of AP-1 to upregulate GFAP expression, resulting in glial scar formation following traumatic sciatic nerve injury [94]. The inhibition of JNK phosphorylation through genetic modification or pharmacological treatment can ameliorate reactive oxygen species (ROS)-induced cell death, prevent inflammatory factor production and enhance electrical conduction in damaged nerves [95]. JNK transcription is required for the activation of NGF-responsive genes to achieve proper development and function during peripheral nerve regeneration [96]. PDGF stimulates angiogenesis, vascular smooth muscle cell migration, axonal outgrowth, regeneration and peripheral target innervation via activation of c-JNK and Akt phosphorylation [97, 98]. FGF-2 has also consistently been shown to induce angiogenesis and enhance neural regrowth, branching and plasticity through the activation of JNK/c-Jun signaling after sciatic nerve transection in adult rats [99, 100]. However, the impact of each specific GF in mediating the JNK/c-Jun pathway during the different stages of nerve development and myelination remains to be elucidated.

### RhoA/ROCK signaling

RhoA/ROCK signaling is regarded as a negative regulator of cytoskeletal reorganization and functional recovery following PNI. For example, excessive activation of this signaling pathway can result in growth cone collapse and neuronal elongation inhibition [101]. In the PNS, myelin-associated inhibitors binding to the neurotrophin receptor p75^NTR^ trigger RhoA/ROCK signal activation, which leads to neurite outgrowth inhibition and restraint of axon regeneration [102]. Blocking the RhoA/ROCK pathway with Y-27362 significantly promotes axonal sprouting and locomotor functional recovery in an optic nerve injury model [103], identifying RhoA/ROCK pathway inhibitors as potential therapeutic options in clinical applications. GDNF promotes neurite outgrowth and neuronal survival by suppressing GDNF family receptor alpha 2 (GFR alpha2)-mediated RhoA activation [104, 105]. The bFGF-mediated regulation of proliferation, migration and morphogenesis during neurite development and regeneration is related to suppressing the RhoA/ROCK signal [106]. In our previous research, we reported that bFGF could inhibit RhoA by activating Ras-related C3 botulinum toxin substrate 1 (Rac1) to reduce neurofunctional deficits [107]. Thus, the inhibition of RhoA/ROCK activation plays important roles in neuroprotection and neurogenesis in nervous system diseases. However, there is a need for a deeper understanding of the exact mechanism of how different kinds of GFs regulate the RhoA/ROCK signaling pathway following PNI.

### Other nonclassic signaling pathways

Recent years have found that signaling pathways, such as neuregulin 1 (Nrg1)-ErbB, Janus kinase (JAK) 1/signal transducer and activator of transcription 3 (STAT 3) and PLCg/Ca^2+^, also participate in axonal guidance and regeneration, neuroprotection, and synaptic plasticity. The activation of the Nrg1-ErbB axis positively regulates SC differentiation and myelin sheath thickness [108]. The decreased expression of Nrg1 in SCs induces abnormally thin myelin sheaths in the PNS of Nrg1 mutant mice, which can be reversed by the injection of soluble FGF10 into the injured sciatic nerve [109, 110]. Additionally, after interacting with p75^NTR^, NGF can directly activate Nrg1-ErbB signaling to regulate axonal sorting and myelination [111, 112]. During nervous system regeneration, JAK/STAT 3 plays a substantial role in controlling cellular proliferation, migration and myelination. Moreover, the JAK/STAT 3 axis is also regarded as a novel therapeutic target for ameliorating nerve injury [113]. The use of a special JAK agonist, 4-methylhistamine dihydrochloride (4-MeH), can result in remarkably improved axon growth and remodeling in DRG [113]. BDNF and NT-3 exert their neurotrophic and neuroprotective effects on traumatic nerve injury through the activation of the JAK/STAT 3 pathway [114, 115]. An additional transduction pathway involving tPLCg/Ca^2+^ has been implicated in the control of cell proliferation and growth, inducing neural crest differentiation and supporting neuronal development by FGF-2 [29]. Overall, signaling pathways involved in GF regulation of peripheral nerve structure and function are increasingly being investigated or discovered. This will provide a potential therapeutic strategy for treating PNI.

## The roles of NGF and FGF-2 in PNI

NGF and PNI. NGF was the first isolated GF, and it consists of two 12.5 kDa polypeptide chains that are in a symmetrical structure [116]. As the most completely characterized GF, NGF shares a high degree of sequence homology with other GFs, including BDNF, NT-3 and NT-4/5. NGF exerts different biological effects by binding its 2 types of receptors: TrkA and p75^NTR^. Functionally, high-affinity TrkA was found to elicit neuronal survival and differentiation after activation by NGF. p75^NTR^, a low-affinity receptor for NGF, is widely expressed in PNS glial cells. Studies have shown that p75^NTR^ overactivation is associated with the induction of nerve apoptosis in experimental animals. Transgenic experiments have demonstrated that silencing the apoptotic p75^NTR^ signal is beneficial for TrkA receptor-mediated neuronal survival in developing sympathetic neurons [117], which emphasizes the importance of p75^NTR^ as a modifier of TrkA to control neuronal fate. NGF, having the double effects of neurotropism and neurogenesis, is associated with binding to the p75^NTR^ and/or TrkA receptor to activate specific downstream intracellular signaling cascades, including MEK/ERK, PI3K/Akt, and JNK/c-Jun [118]. Following PNI, NGF expression begins to increase within the first day at the lesion sites. This upregulated trend in NGF level peaks within 7 days post injury, remains high until the 14th day, and subsequently began to decrease until day 30 [119]. These different styles of endogenous NGF expression indicate that NGF plays a vital role in neuroprotection and neurorestoration during nerve regeneration. Previous studies have shown that NGF can reverse axonal degeneration, demyelination, and atrophy in diabetic neuropathy, and the efficacy of recombinant human NGF (rhNGF) has been tested in a phase III trial of patients with diabetic neuropathy [120]. Recently, our groups have found that the administration of NGF significantly decreased the time needed for regrowth and remyelination after PNI, which was closely associated with NGF accelerating the collapse of degenerative nerves and promoting myelin debris clearance. Furthermore, we also revealed that these effects of NGF were likely mediated by p75^NTR^/AMP-activated protein kinase (AMPK)/mammalian target of the rapamycin (mTOR)-dependent pathways, resulting in enhanced autophagic activities in SCs [121]. Thus, these discoveries provide strong support that NGF may serve as a powerful pharmacological therapy for peripheral nerve injuries.

In vitro, NGF has been found to promote survival, proliferation and neurite outgrowth in sensory and sympathetic neurons [122]. Histological and morphometric studies have revealed that NGF treatment notably increased the number and diameter of myelinated nerve fibers and accelerated electrophysiological parameters after sciatic nerve crush [123]. However, NGF facilitation of peripheral nerve regeneration must satisfy the following requirements: (a) the administration of exogenous NGF must reach a certain amount that could influence the glial and neural response to injury; (b) NGF-activated downstream signaling pathways should be able to influence the intracellular biological effect that resisted or reversed insults and injury; (c) the dose of NGF should elicit neuroprotective and neurotrophic effects; and (d) insufficient or absent NGF supplementation should be involved in increasing apoptosis and vulnerability to injury. Thus, the team of Kemp revealed that the administration of NGF at 80 ng/day for 3 weeks was the optimal dose of NGF provide saturated binding of TrkA and reach successful regeneration following PNI [124]. NGF-induced peripheral nerve regeneration and neurite outgrowth after PNI depend on gp130/STAT3 signaling activation [125]. However, considering that nerve regeneration is a dynamic process that requires a certain amount of GFs over a long period of time, the use of engineered materials delivering GFs may be an ideal strategy for supporting axon regeneration. The gradient of NGF-immobilized nanofibrous nerve conduits significantly guided DRG neurite outgrowth and enhanced morphological and functional recovery in a rat 14 mm sciatic nerve defect model, and the effect was similar to that of the autograft [126]. A longitudinally oriented collagen conduit (LOCC) loaded with NGF provided a preferential environment for functional and morphological recovery after sciatic nerve transection [127]. The combined delivery of NGF and GDNF with an artificial nerve conduit scaffold provided synergistic biological action on axonal elongation [128]. Thus far, the testing of therapies that combine GFs with delivery systems may offer a promising strategy for sensory and motor innervation in nerve defect models.

FGF-2 and PNI. FGF-2, also known as bFGF, is regarded as the most important molecule for promoting nerve regeneration among the 23 members of the FGF family [129]. FGF-2 is a mitogenic cationic polypeptide containing 155 amino acids, and it has a molecular weight of 16 ~ 18.5 kDa. FGF-2 is widely expressed in the pituitary, brain and nerve tissue, and it plays an important role in mitogenesis and differentiation during embryonic development. Moreover, some FGF-2 isoforms also have important effects in mediating SC or oligodendrocyte proliferation, differentiation and myelination after injury to the central or peripheral nervous system. There are four subfamilies of tyrosine transmembrane receptors in vertebrates (FGFR1 to 4) that bind FGF-2 with different affinities. In peripheral nerves, endogenous FGF-2 produced by SCs or neurons can gather in the intracellular domain to interact with intracellular substrates and activate special signal transduction molecules through binding with its corresponding receptors. These downstream signaling pathways that may be employed by FGFRs include Ras/MAPK/Erk, PI3K/Akt, and PLCg/Ca^2+^ [130]. Meisinger et al. found that the expression of both FGF-2 protein and its mRNA occurred as early as 5 h after a nerve crush injury, and this upregulated trend lasted for at least 4 weeks in the damaged nerve stump [131, 132]. In vitro and in vivo studies of the PNS showed that the exogenous FGF-2 isoform mediated neurotrophic activities, stimulated SC survival and accelerated myelin debris clearance at the early stage of peripheral nerve lesion. Local injection of FGF-2 into the lesion region markedly ameliorated structural and functional recovery in the PNI model, protecting the survival of SCs and promoting axon and myelin regeneration.

Many investigations have attempted to reveal the molecular mechanism by which FGF-2 regulates sensory and sympathetic growth and nerve regeneration. In a study by Gu et al., FGF-2 induced the differentiation of neural stem cells towards SCs. Moreover, they also confirmed that this effect involved the activation of the MAPK/ERK signaling pathway by FGF-2 binding to FGFR1 [133]. In our previous research, we found that FGF-2 could also effectively ameliorate neural survival and functional recovery in SCI and ischemic oxidative injury through PI3K/Akt/mTOR or ERK1/2 signaling-mediated autophagy or suppression of ER stress [85, 134]. In the process of nerve regeneration, FGF-2 does not simply act on its own. FGF-2 also interacts with other GFs in lesion regions to promote multiple biological effects. Seki et al found that the application of FGF-2 and IGF in different ratios resulted in different degrees of rescued growth cone collapse in sensory neurons [135]. In addition, the administration of exogenous FGF-2 benefited interleukin-6 and its related receptor expression in differentiated and proliferating SCs, respectively [136]. To enhance the bioactivity of FGF-2, filling chitosan nerve guides or collagen scaffolds with GFs can provide a promising strategy for repairing peripheral nerve defects. A cellular neurotrophic factor delivery system made of chitosan and NVR-hydrogel can maintain FGF-2 bioactivity and control its release for more than 3 months. Moreover, these chitosan/NVR-Gel filling FGF-2 conduits enhanced neurite outgrowth at certain distances, increased axon and myelin numbers, and promoted the recovery of sensory and motor function in a rat model with 10 mm gap repair [137]. Collagen scaffolds modified with FGF-2 and CNTF were observed to improve peripheral nerve reconstruction through electrophysiology assessment and histological examination in a long facial nerve gap model [138]. Such nerve conduits synthesized by different kinds of biomaterials delivering FGF-2 and elucidating how they modulate the cellular response to facilitate peripheral nerve regeneration are an exciting direction for future research.

## Conclusion and future perspectives

In this review, we mainly discussed the role of the GF and complexes of GF-materials in peripheral nerve regeneration. Moreover, we selected certain GFs to reveal their related molecular mechanisms. We can conclude that each GF is able to bind to its respective receptor to exert its biological effects by activating different kinds of downstream signaling pathways. Although GFs possess the capability of enhancing the survival of SCs and promoting axon regrowth and remyelination after PNI, there are many shortcomings of GFs that must be overcome to optimize their properties and achieve the best therapies for axon regeneration and functional recovery. The employment of engineering materials incorporating multiple GFs is regarded as the most promising strategy for maintaining GF bioactivity because these delivery systems not only facilitate the controlled spatiotemporal release of encapsulated GFs but also locally deliver GFs to the target region at a safe dosage. Biocompatible and biodegradable nerve conduits integrating a variety of GFs have been widely used in animal trials, and some of these are being applied in clinical practice. Therefore, localized delivery of several exogenous GFs into the conduit lumen may provide a suitable microenvironment for regenerating axons towards the correct target and enhancing peripheral nerve reconstruction.
